# Using conditional inference to quantify interaction effects of socio-demographic covariates of US COVID-19 vaccine hesitancy

**DOI:** 10.1371/journal.pgph.0001151

**Published:** 2023-05-12

**Authors:** Ke Shen, Mayank Kejriwal

**Affiliations:** Information Sciences Institute, University of Southern California, Marina del Rey, Marina del Rey, California, United States of America; University of Colorado Denver - Anschutz Medical Campus: University of Colorado - Anschutz Medical Campus, UNITED STATES

## Abstract

COVID-19 vaccine hesitancy has become a major issue in the U.S. as vaccine supply has outstripped demand and vaccination rates slow down. At least one recent global survey has sought to study the covariates of vaccine acceptance, but an inferential model that makes simultaneous use of several socio-demographic variables has been lacking. This study has two objectives. First, we quantify the associations between common socio-demographic variables (including, but not limited to, age, ethnicity, and income) and vaccine acceptance in the U.S. Second, we use a conditional inference tree to quantify and visualize the interaction and conditional effects of relevant socio-demographic variables, known to be important correlates of vaccine acceptance in the U.S., on vaccine acceptance. We conduct a retrospective analysis on a COVID-19 cross-sectional Gallup survey data administered to a representative sample of U.S.-based respondents. Our univariate regression results indicate that most socio-demographic variables, such as age, education, level of household income and education, have significant association with vaccine acceptance, although there are key points of disagreement with the global survey. Similarly, our conditional inference tree model shows that trust in the (former) Trump administration, age and ethnicity are the most important covariates for predicting vaccine hesitancy. Our model also highlights the interdependencies between these variables using a tree-like visualization.

## Introduction

As of January 27, 2022, more than 71.8 million people in the United States had reported being infected with Coronavirus disease 2019 (COVID-19), a highly contagious viral illness caused by SARS-CoV-2 [1]. To protect against the continued spread of the SARS-CoV-2 virus, COVID-19 vaccines were approved in late 2020 and early 2021 for public use in countries across the world. In May 2021, President Joe Biden announced his goal as getting at least 70% of Americans partially vaccinated against COVID-19 by early July at the latest. However, government records indicate that this goal was finally achieved only in November 2021, not due to supply constraints, but rather, due to vaccine hesitancy among certain segments of the population [2, 3]. As late as September 2022, more than 31% of Americans are still not fully vaccinated. In order to speed up vaccination and reduce hesitancy, the use of material incentives and communication-based outreach are being explored. While laudable, for maximal effectiveness, we argue that such efforts need to carefully identify vaccine-hesitant subgroups in the U.S. and investigate the most important predictors of vaccine hesitancy. For such identification to be accurate and fine-grained, while still preserving privacy, it is important to understand the interaction and conditional effects between relevant socio-demographic correlates that are known to be important predictors [4]. An example of a conditional effect is captured in a (illustrative-only) statement such as ‘People between 18–25 tend to be vaccine hesitant, but the hesitancy disappears if the education exceeds college level.’

Several studies have highlighted key factors associated with vaccine hesitancy in different countries, and considerable heterogeneity has been observed. Lazarus et al. provided a global survey of COVID-vaccine acceptance, with the study suggesting that men were less likely to get vaccinated than women [5]. People with higher incomes and education were also more vaccine accepting. However, research into vaccine hesitancy in the UK suggested higher vaccine hesitancy among women, younger age groups and those with lower education levels [6, 7]. Minority groups, including Black individuals, were also found to be more hesitant. Similar results were shown for Portugal, including individuals who had lost income during the pandemic and had lower confidence in the vaccine [8]. In Arabic countries, researchers reported that male respondents with higher educational levels, and those with histories of chronic disease, were more COVID-vaccine accepting [9].

Vaccine hesitancy has been documented as a challenge several times in previous research [3, 6, 7, 10–17]. Coustasse, Kimble and Maxik revealed that layperson skepticism may have been compounded by political influences during the Donald Trump presidency [10]. Callaghan et al. explained that the two most cited reasons for vaccine hesitancy are concerns about vaccine safety, as well as lack of trust in vaccine effectiveness [3]. Some researchers have also focused on special groups of people, such as medical students and healthcare workers [18, 19]. Differences in ethnicity have been frequently mentioned in vaccine hesitancy research [10–16, 20]. In work resembling this study, multiple regression analyses have been used to caution the government into paying more attention to vaccine hesitant groups, who (in the U.S., at least) are often minority groups who have had negative experiences with the healthcare system in the past, including African-Americans and Hispanics [11, 13, 20]. Individuals with conservative political affiliations (including Republicans in the U.S.), and those with children at home, have also been found to be vaccine hesitant [21].

Given this body of prior work, an important motivation for the public health community is to understand the influence, and relevance, of socio-demographic variables (with examples including gender, age and ethnicity) on vaccine acceptance. A non-comprehensive set of examples of such studies include [22–26].

We note that such studies cannot always be conducted using aggregate data alone, since we need to observe the socio-demographic variables and vaccine hesitancy (even if self-reported) at an *individual* level. Subsequently, we discuss the details of the Gallup COVID-19 survey that allowed us to conduct a retrospective analysis using individual-level data. Our analysis is designed with two objectives in mind. First, we aim to quantify the associations between common socio-demographic variables and vaccine acceptance specifically in the U.S. Second, we aim to quantify and visualize the interaction and conditional effects of relevant socio-demographic variables that are known to be important correlates of vaccine acceptance in the U.S., on vaccine acceptance itself.

Although our objectives are aligned with those of the studies cited earlier [22–25], the survey data used spans a representative sample of communities, ages and income across the entire United States, instead of focusing primarily on youth (such as in [24]) or on a different country, such as Italy [23]. Objective 1 of our study is most similar to an early global study [5] that heavily relied on univariate regressions to quantify associations, but as we show in our results, there are some key points of disagreement between our U.S.-based conclusions and those of the global populace. This is not surprising, but adds more fine-grained insight to these earlier studies. In structure, our study is also similar to [22, 25], the former studying the issue by taking into account past experience of racial discrimination, socio-demographics and co-morbidity. However, they did not study interaction effects among variables by using an advanced statistical model such as a conditional inference tree, and their study sample was smaller than ours by a factor of 15x (2,650 respondents in [22] versus 16,322 in ours, polled by the Gallup organization that operates independently of the authors).

In contrast, the authors in [25] study the vaccine hesitancy issue by considering factors like confidence, circumspection, and complacency. They also consider spatio-temporal effects, similar to one of our own experiments in this paper. However, to our knowledge, the proposed study is the first one to use a conditional inference tree model to understand the interaction effects of socio-demographic covariates on U.S. vaccine hesitancy by also using the conditional inference tree model. Compared to the previous study, our study emphasizes not only the significant effects of socio-demographic covariates on U.S. vaccine hesitancy, but also the complex interdependencies between these socio-demographic characteristics of the population. By using recursive partitioning, and applying statistical significance tests at each partition, the conditional inference tree model is able to quantify these interdependencies in a statistically robust manner.

## Materials and methods

### Survey participants, data collection and cleaning

We begin this section by describing Gallup’s study design, on the basis of which our retrospective analysis was conducted. Gallup [27] first began fielding its COVID-19 questions on March 13, 2020 by launching a specific web survey that collected people’s responses during the COVID-19 pandemic, polling daily random samples of the Gallup Panel, which is a probability-based, nationally representative panel of the U.S. adults. The obtained samples were weighted by Gallup to correct for the non-response bias. The adjustments were made to ensure that the non-response samples match the national demographics of gender, age, race, Hispanic ethnicity, education and region. The demographic weighting adjustments are based on the latest Current Population Survey figures for the aged-18-and-older U.S. population. The total number of observations after the adjustments in this survey data is 141,119. More details about the Gallup sampling process have been included in S1 Text.

As in other Gallup surveys, such as the Gallup World Poll [28], questions measuring employment, health, and demographics are included. We reproduce the exact wording of the questions asked by Gallup to obtain data per socio-demographic variable in S1 Table. The vaccine acceptance question was asked in Gallup Panel COVID-19 Web Survey starting from July 20, 2020:

‘If an FDA-approved vaccine to prevent coronavirus/COVID-19 was available right now at no cost, would you agree to be vaccinated?’

A possible response of ‘Yes’ or ‘No’ polls a person’s willingness to be vaccinated. Since the ‘vaccine acceptance question’ was added on July 20, the responses of interest in this study are from July 20, 2020 to Jan 1, 2021, with 29,399 valid responses collected across this time period. Since there were questionnaire changes (i.e., some new questions were added and some old questions were removed in an update of the survey), some participants were not asked to answer some questions. For example, the vaccine acceptance question was asked from July 20, 2020 to Jan 1, 2021, and the ‘trust-in-Trump-administration’ question (discussed in the next section) was asked from July 20, 2020 to Sep. 14, 2020; hence, responses for the trust-in-government question after Sep. 14, 2020 will be encoded as ‘NaN’ (Not a Number). Indeed, Gallup encoded the responses as NaN if the participants did not provide any data points for the survey questions. In the survey, this question imports 11,783 NaN values in the 29,399 responses because these 11,783 participants did not answer this question. After filtering records that include NaN in one or more values, 16,322 instances were left.

The vaccine acceptance before removing NaN values was measured to be 70.6%, and after NaN filtering, as described above, the vaccine acceptance remained at similar levels (71.17%), consistent with what has been reported in the press and in other US-based surveys [2, 3]. Our investigation adhered to the STROBE guidelines for the reporting of observational studies (see S4 Table).

### Ethics statement

The study, with IRB number of UP-21-00692, was submitted on 8/10/2021 and approved by the University of Southern California Institutional Review Board on 9/29/2021. The study is retrospective and utilized data already collected by Gallup, which obtained consent from all participants whose data was used for this research.

### Analysis

In the filtered survey data (n = 16,322), women comprised 45.5% of the study population. 50.7% of all participants were in a household that earned more than 90,000 US$ per year. About 91% had more than a high school education, and 58.4% of the respondents were in age range of 25–64 years old. 42.47% participants were employed full-time. White individuals constitute the majority (87%) of respondents. Around 90% of respondents obtained at least a high school diploma, and 41.12% of respondents were over 65 years old. Democrats were 41.80% of participants, and Republicans were 25.15% (the remainder were independents). We verified that the proportions of different demographic groups in the data points are close to the latest U.S. Census results [29].

When describing the survey data in the previous section, we mentioned a ‘trust-in-Trump-administration’ question. This question probed participants on whether they trusted the Trump administration during the COVID-19 pandemic and is worded as follows: ‘Please think about the recent impact of the coronavirus (COVID-19) on your life when responding to the following and indicate your level of agreement or disagreement: I have confidence in the leadership of President Donald Trump to successfully manage emerging health challenges’ Responses to this question were recorded on a five-point scale, from strongly disagree (1) to strongly agree (5).

Values on this scale greater than a threshold of 3 were used to separate individuals who trusted in the Trump administration versus those who did not. 34.94% of respondents reported high levels of trust in the administration. A summary of valid responses to the survey questions and the characteristics of these respondents are provided in S2 Table.

We also provide the frequency (or proportion) of vaccine acceptance in each socio-demographic group in Table 1. For instance, among respondents with a household income between 0 and $36,000, 66.6% are vaccine-accepting. A full statistical profile of respondents’ answers per covariate level is reproduced in S2 Table. In response to the vaccine question, 71.2% of respondents (11,616 of 16,322) would agree to be vaccinated, or have already received a COVID-19 vaccine. Of these 11,616 individuals, 5,096 women and 6,520 men were willing to be freely vaccinated. Comparing White individuals to minorities, 72.4% White individuals had positive responses, compared to 62.2% non-White respondents.

**Table 1:** The percentage of respondents who responded positively to the vaccine acceptance (VA) question in each socio-demographic group. The heading of each sub-table is the socio-demographic variable, with each column in the sub-table corresponding to a level of the variable. Each cell value contains the percentage of respondents within that level who responded positively to the vaccine acceptance question. For example, in the *Gender* sub-table, 73% of male participants responded positively to the vaccine acceptance question compared with 69% of female respondents. These results are based on the Gallup Panel COVID-19 Web Survey, which began fielding on March 13, 2020 (and last updated in our copy of the dataset on Feb 22, 2021) with daily random samples of the U.S. adults, aged 18 and older who are members of the Gallup Panel.

	**Annual household income**							
	*[0*, *36*,*000)*	*[36*,*000*, *60*,*000)*	*[60*,*000*, *90*,*000*	*[90*,*000*, *120*,*000)*	*[120*,*000*, *180*,*000)*	*[180*,*000*, *240*,*000)*	*[240*,*000*, *∞)*	
VA	66.6%	68.5%	69.9%	70.5%	73.4%	76.3%	79.6%	
	**Age**							
	*18–24*	*25–40*		*41–54*		*55–64*		*65+*
VA	78.0%	71.9%		62.2%		66.9%		77.9%
	**Race/Ethnicity**							
	*White*	*Hispanic*		*Black*		*Asian*		*Other*
VA	72.4%	62.4%		52.4%		75.9%		66.0%
	**Political Party**							
	*Democrat*		*Republican*		*Independent*		*Other*	
VA	85.4%		55.8%		68.2%		52.4%	
	**Employment Status**							
	*Full-time*		*Part-time*		*Involuntary unemp*.		*Not in labor force*	
VA	68.1%		67.8%		65.2%		75.1%	
	**Gender**							
	*Male*				*Female*			
VA	73.3%				68.6%			
	**Education**							
	*Less than a high school diploma*	*High school graduate*	*Technical*, *trade*, *or business school or program after high school*	*Some college*, *college*, *university*, *or community college—but no degree*	*Four year bachelor’s degree from a college or university*	*Some postgraduate or professional schooling after graduating college*, *but no post*	*Two year associate degree from a college*, *university*, *or community college*	*Postgraduate or professional degree*, *including master’s*, *doctorate*, *medical*, *or law*
VA	42.4%	61.3%	57.2%	64.1%	73.5%	75.8%	65.7%	81.5%
	**Trust in Trump administration**							
	*No*				*Yes*			
VA	81.8%				51.3%			

### Univariate regression and odds ratios

To study the relative influence of socio-demographic characteristics in vaccine acceptance and facilitate comparisons with the survey results by Lazarus et al., which is our first objective, we analyzed the distribution of the vaccination responses against similar demographic variables (such as age, gender, income and level of education) and other covariates of interest for all 16,322 instances in the dataset [5]. In their work, Lazarus et al. had surveyed 13,426 people in 19 countries, such as the United States, China and Russia, to determine potential COVID-19 vaccine acceptance rates, and factors influencing this rate. They found that respondents from China gave the highest proportion of positive responses when asked if they would take a ‘proven, safe, and effective vaccine’, whereas Russian respondents gave the lowest proportion of positive responses. They also found that, globally, older people were more likely to accept the COVID-19 vaccine. Gender-specific vaccine acceptance differences were small, with men slightly less likely to respond positively than women. Also, higher income and higher level of education were both associated positively with vaccine acceptance. Finally, Lazarus et al. found that, if an individual trusted their government, they were more likely to respond positively to their employer’s vaccine recommendation than someone who did not.

Similar to Lazarus et al. [5], we consider seven independent variables in the regression analyses: annual household income, age, ethnicity, political party, employment status, gender, level of education, and trust in the (then current) Trump administration. Details about these covariates are provided in S2 Table. Note that one reason we aim to compare our own results to those of Lazarus et al. using a similar methodology is to verify that the findings about COVID-19 vaccine hesitancy are robust and reasonably stable between the time that the two sets of analyses were conducted.

Specifically, we compute a set of univariate logistic regressions, defining the label as 1 if a respondent answered ‘Yes’ in response to the vaccine question, and 0 if the response was ‘No’. These labels (encoded as 0 or 1) are used as the values of the dependent variable in the regressions. They also represent the outcomes measured in the odds ratio (OR) study. Each univariate logistic regression yields an OR. As mentioned by Szumilas [30], the OR is a measure of association, with the odds of an outcome defined as the ratio of the probability that the outcome occurs, to the probability that the outcome does not occur. The OR then represents the odds that an outcome will occur given a particular exposure to the variable of interest, compared to the odds of the outcome occurring in the absence of that exposure. When a univariate regression is calculated, the regression coefficient of the categorical variable is the estimated odds ratio of the outcome, given the corresponding exposure.

The interpretation of an OR greater than 1 is that there is a greater likelihood that the particular socio-demographic subgroup (used as predictor in the regression) will accept the vaccine, *compared* to the chosen reference for that variable. Hence, the OR is a relative measure. The demographic variables for which we independently compute ORs include age, gender, ethnicity, party, employment status, income, and education. The univariate regression also provides a *p*-value for each odds ratio to measure whether the odds ratio between the test category and the reference category is significantly different from 1.

Furthermore, based on the sum of ORs, we could give a descriptive score for each respondent’s willingness to be vaccinated. To be specific, the value of the anchor choice (the ‘reference’) for each socio-demographic variable is set to 1 (e.g., ‘White’ in *Race/Ethnicity* and ‘full-time’ in *Employment Status*); values of all other choices for a given survey question are represented by their ORs when regressed against the corresponding anchor choice (for example, 0.74 for ‘Hispanic’ in *Race/Ethnicity*; see Table 1). The sum of these ORs for a given individual suggests that the higher the score, the higher the odds of the individual accepting COVID-19 vaccination. As we discuss in a subsequent section, these numbers can potentially help devise communication-based outreach and other policies for reducing vaccine hesitancy among subgroups where the hesitancy is particularly high.

### Conditional inference tree

To explore the conditional effects between relevant socio-demographic correlates on vaccine acceptance, we used an established statistical classification model called a *conditional inference tree (CIT)*, which uses unbiased recursive partitioning of dependent variables (predictors) based on observed correlations at the individual level [31, 32]. Similar to the univariate regressions, the responses to the vaccine question are treated as outcome responses in the CIT model.

CITs embed tree-structured regression models into a well-defined theory of conditional inference procedures. It is unnecessary to consider data imputation techniques since the models generate high prediction performance. The technique is suitable for non-parametric data (e.g., nominal and binary), including nominal variables with many levels (as are often observed with ethnicity and employment status variables in survey-based studies such as this one). A CIT tree uses a unified unbiased splitting technique (called permutation-based significance tests) based on *p*-values for variable selection and pruning. making it more statistically robust than classification and regression trees (CARTs) [33]. The null hypothesis used in the CIT variable selection is that the distribution of response (the dependent variable) is equal to the distribution of the conditional distribution of the response given specific covariate. The CIT model achieves greater statistical robustness by first testing the global null hypothesis between each predictor and the response variable, and selecting the predictor for which the null hypothesis can be rejected most confidently (i.e., with the lowest *p*-value). Then, the model performs a binary split on the selected predictor. These two steps are recursively performed, until the null hypothesis cannot be rejected at a minimum pre-specified confidence level (set to 90% in our results) for any remaining predictor.

Compared to logistic regression models, CITs provide us with more interpretability and flexibility, while still maintaining high prediction accuracy [34]. As we show subsequently, they can also be visualized, in direct support of our second objective. This also makes it a superior alternative to methods like logistic regression for understanding *conditional* effects. For instance, while the logistic regression model fits a single linear boundary to divide samples into *two* groups, the CIT model bisects the sample space into progressively smaller regions using statistical significance tests. CITs are therefore better able to capture the non-linear or non-monotonic relationships between predictors and dependent variables. Another strength of CITs is that they tend to be robust to noisy data (outliers) and can handle categorical variables more naturally (i.e., without requiring special encoding of any categorical features into dummy or indicator variables). The model has been applied in a similar context in the past to study conditional effects with respect to climate change skepticism [35].

The R package *party* is used for our recursive partitioning analysis, with the max depth of CIT set to 4, and with the *p*-value required to be less than 0.01, in order for a node to be split by the model. Note that the variable *age* in CIT model is treated as a numeric variable rather than categorical variable, as in the univariate regression. The performance of the CIT model is evaluated by its prediction accuracy.

## Results

### Relative influence of socio-demographic variables

Table 2 reports results using univariate regressions, including odds ratios, for positive responses to the vaccine question using eight variables, including whether or not the respondent had confidence in the leadership of the Trump administration to successfully manage emerging health challenges.

**Table 2:** Univariate regression outputs for vaccine acceptance against demographic variables and other covariates of interest (with reference indicated as ‘Compared to…’). For each covariate, we show its odds ratio (OR), 95% confidence interval and the corresponding *p*-values. These results are based on the Gallup Panel COVID-19 Web Survey, which began fielding on March 13, 2020 (and last updated in our copy of the dataset on Feb 22, 2021) with daily random samples of the U.S. adults, aged 18 and older who are members of the Gallup Panel.

	**Annual household income (Compared to [0, 36,000))**						
	*[36*,*000*, *60*,*000)*	*[60*,*000*, *90*,*000*	*[90*,*000*, *120*,*000)*	*[120*,*000*, *180*,*000)*	*[180*,*000*, *240*,*000)*	*[240*,*000*, *∞)*	
OR	1.09	1.16	1.20	1.38	1.61	1.96	
95% CIs	(0.96, 1.23)	(1.03, 1.31)	(1.06, 1.35)	(1.22, 1.56)	(1.37, 1.90)	(1.66, 2.31)	
*p*-value	0.17	0.01	0.00	0.00	0.00	0.00	
	**Age (Compared to 18–24)**						
	*25–40*	*41–54*		*55–64*		*65+*	
OR	0.72	0.46		0.57		1.00	
95% CIs	(0.42, 1.23)	(0.27, 0.78)		(0.33, 0.96)		(0.58, 1.67)	
*p*-value	0.228	0.00		0.04		0.97	
	**Race/Ethnicity (Compared to White)**						
	*Hispanic*	*Black*		*Asian*		*Other*	
OR	0.74	0.42		1.20		0.63	
95% CIs	(0.64, 0.86)	(0.36, 0.49)		(0.91, 1.59)		(0.47, 0.86)	
*p*-value	0.00	0.00		0.20		0.00	
	**Political Party (Compared to Democrat)**						
	*Republican*			*Independent*		*Other*	
OR	0.22			0.37		0.19	
95% CIs	(0.20, 0.24)			(0.33, 0.40)		(0.15, 0.23)	
*p*-value	0.00			0.00		0.00	
	**Employment Status (Compared to Full-time)**						
	*Part-time*			*Involuntary unemp*.		*Not in labor force*	
OR	0.98			0.88		1.41	
95% CIs	(0.87, 1.11)			(0.74, 1.04)		(1.31, 1.52)	
*p*-value	0.79			0.14		0.00	
	**Gender (Compared to male)**						
	*Female*						
OR	1.26						
95% CIs	(1.18, 1.35)						
*p*-value	0.00						
	**Education (Compared to less than a high school diploma)**						
	*High school graduate*	*Technical*, *trade*, *or business school or program after high school*	*Some college*, *college*, *university*, *or community college—but no degree*	*Four year bachelor’s degree from a college or university*	*Some postgraduate or professional schooling after graduating college*, *but no post*	*Two year associate degree from a college*, *university*, *or community college*	*Postgraduate or professional degree*, *including master’s*, *doctorate*, *medical*, *or law*
OR	2.15	1.82	2.43	3.77	4.26	2.6	5.99
95% CIs	(1.27, 3.65)	(1.06, 3.10)	(1.44, 4.09)	(2.24, 6.35)	(2.50, 7.25)	(1.54, 4.40)	(3.55, 10.10)
*p*-value	0.00	0.03	0.00	0.00	0.00	0.00	0.00
	**Trust in Trump administration (Compared to No)**						
	*Yes*						
OR	0.23						
95% CIs	(0.22, 0.25)						
*p*-value	0.00						

The table shows that people aged 41–54 are far less likely to accept the vaccine than those aged 18–24, with odds ratio (OR) of 0.46 and 95% confidence interval (CI) of (0.27, 0.78). People aged over 65 are as willing to be vaccinated as youth (18–24), with OR of 1.0 and 95% CI of (0.58, 1.67). In other words, within the United States, older people are more vaccine hesitant relative to youth (compared to global data collected earlier last year); and worryingly, the middle spectrum of the age distribution is (or has become) much more vaccine hesitant compared to global counterparts.

The data also suggests that men are more vaccine-accepting than women, with an OR of 1.26 and 95% CI (1.18, 1.35). Concerning ethnicity, we find that, except for Asians, minorities, including Hispanic and Black individuals, are less likely to accept vaccination compared with White individuals. Black individuals, in particular, are vaccine hesitant (OR = 0.42; 95% CI (0.36, 0.49)), compared to White individuals.

An obvious trend can be seen with annual household incomes: compared to the lowest income group, people at higher household income levels are more likely to be vaccine accepting and the strongest difference (OR = 1.96; 95% CI (1.66, 2.31)) is observed in participants with over $240,000 household income per year, as compared to those making (at most) $36,000 annual household income. Higher levels of education were also associated positively with vaccine acceptability. An OR of 5.99 (95% CI (3.55, 10.10)) was observed for postgraduate participants relative to those with less than a high school education. People not in the labor force (full time students, retired people, and those who are unemployed, but not looking for a job) are also more likely to accept the vaccine (OR = 1.41, 95% CI (1.31, 1.52)), which is consistent with the odds ratio of 65+ participants’ vaccine acceptance (compared to people in the 18–24 age range), since many individuals not in the labor force would, in all likelihood, be categorized into these two age groups. However, we also found that people with a part-time job, or with an involuntary unemployed status, are less likely to positively respond to vaccination. This association is a potential cause for concern, but its underlying reasons are less clear. For instance, it is not possible to tell from the survey data whether these individuals are vaccine hesitant due to their being too busy working (or looking for a job), or due to intrinsic concerns about the vaccine itself. Further research is needed to tease apart the causal underpinnings of this finding.

Concerning political affiliation, Democrats are most inclined to vaccinate, while Republicans, Independents and also those from the third party are more likely to negatively respond to the vaccine question. Those who believed that the Donald Trump government could successfully manage emerging health challenges were less willing to be vaccinated, compared to those who doubted the administration’s COVID-19 policies (OR = 0.23, 95% CI (0.22, 0.25)). These sentiments may have shifted since the new administration was elected, which a follow-up study could aim to quantify.

**Fig 1:**
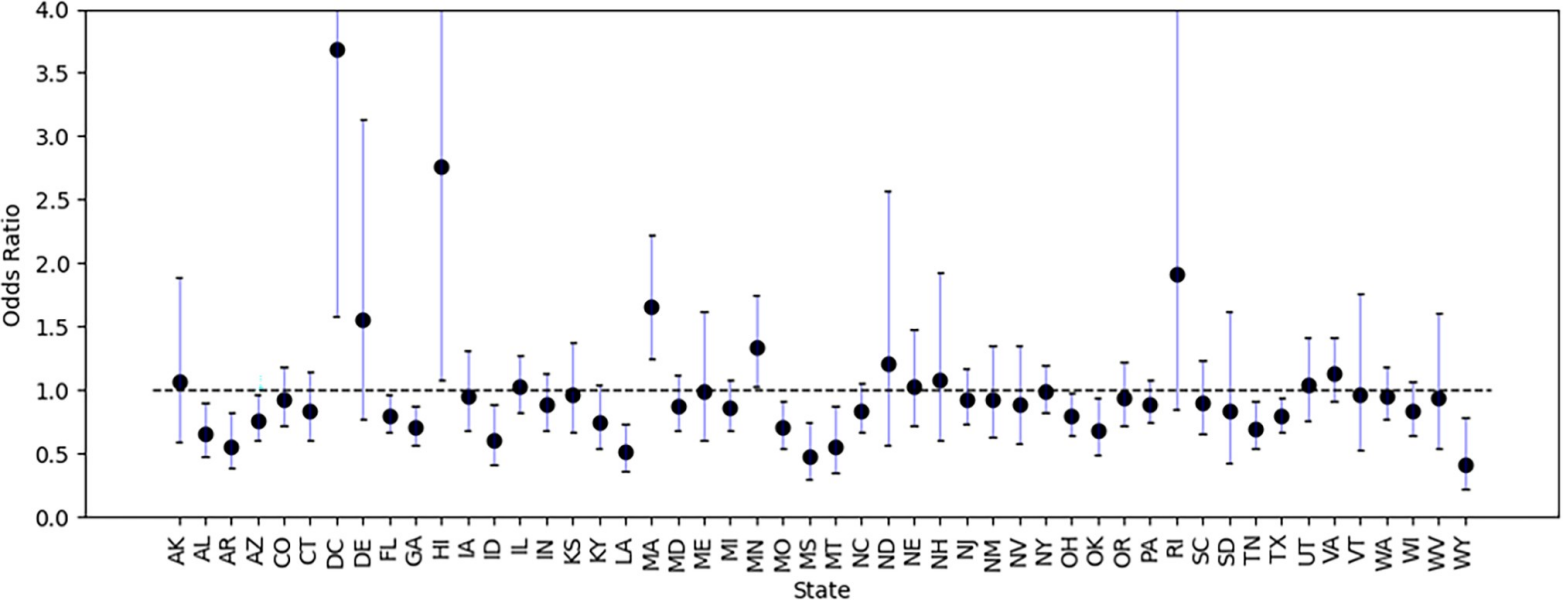
Univariate regression outputs for vaccine acceptance by state, with California as the reference (i.e., with an odds ratio of 1.0). The odds ratio (OR) values are shown as black dots and 95% confidence intervals (CIs) are represented by the vertical bar. These results are based on the Gallup Panel COVID-19 Web Survey, which began fielding on March 13, 2020 (and last updated in our copy of the dataset on Feb 22, 2021) with daily random samples of the U.S. adults, aged 18 and older who are members of the Gallup Panel.

We also report the ORs by state in Fig 1 (with the raw data underlying the figure reproduced in S3 Table), with California as the reference. In the Gallup data, 73.38% of participants from California were willing to accept Covid-19 vaccine, higher than the overall vaccine acceptance (71.17%) in the survey. Comparing other states to California, we found that Democratic states such as District of Columbia (DC), Hawaii (HI), Delaware (DE), Massachusetts (MA), Minnesota (MN), and Rhode Island (RI) are more vaccine-accepting states. The participants from Alabama (AL), Arkansas (AR), Arizona (AZ), Florida (FL), Georgia (GA), Louisiana (LA), Maryland (MD), Mississippi (MS), Montana (MT), Oklahoma (OK), Tennessee (TN), Texas (TX), and Wyoming (WY) were less likely to accept vaccine with respect to the participants from California. Most of these states are Republican states except Maryland. District of Columbia and Wyoming were the two states with the highest and lowest vaccination acceptance, respectively. These results suggest that spatial variables like state can play an important role in explaining vaccine hesitancy. Besides, the results of ORs by state still suggest that the vaccine acceptance is closely related to the political affiliation. In the future, researchers could also consider more advanced geospatial models that account for spatial heterogeneity and local effects that could explain some of these relationships. Encouragingly, recent work has already started to consider this issue in more depth [25, 36, 37].

### Conditional effects between socio-demographic variables

**Fig 2:**
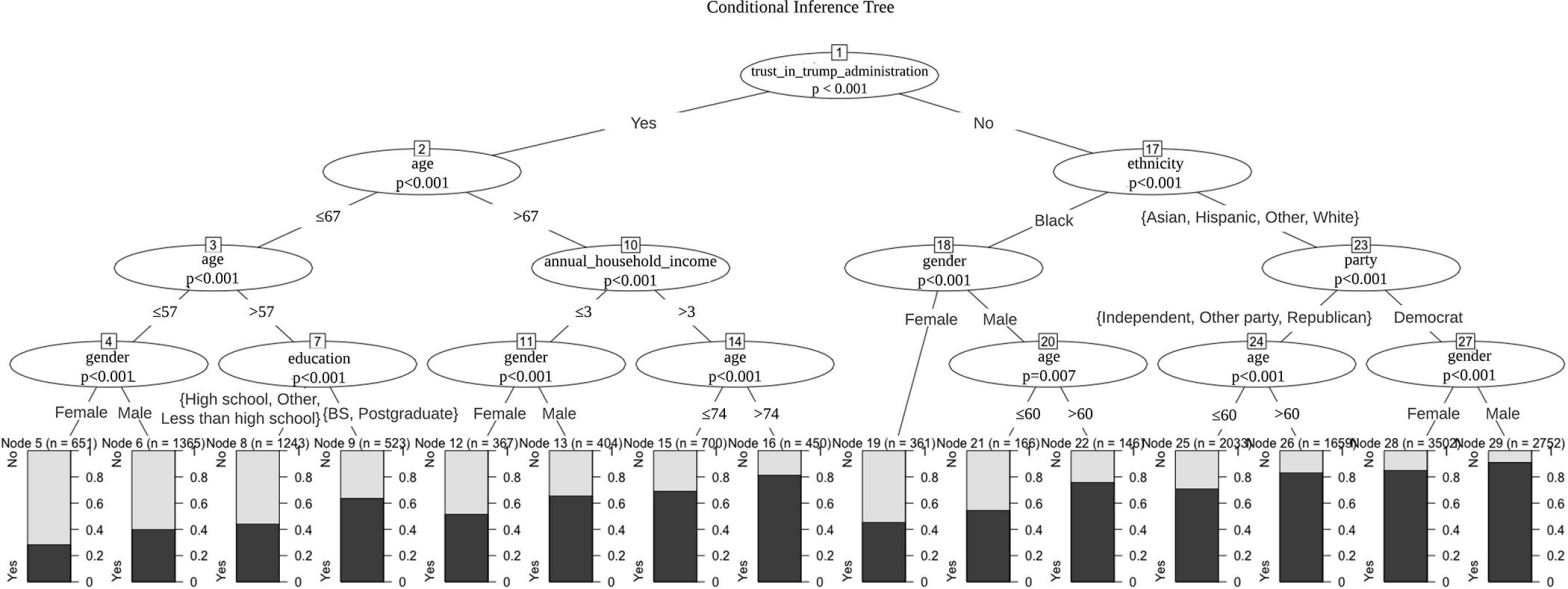
The conditional inference tree (CIT) model for predicting vaccine acceptance. The education node shows the highest academic degree obtained by participants, with ‘BS’ representing a four year bachelor’s degree from a college or university. The tree is four levels deep and shows only statistically significant variables. Total sample size (N) for the vaccine acceptance poll is 16,322. Note that the annual_household_income ≤ 3 represents the participants with less than $90,000 annual household income. These results are based on the Gallup Panel COVID-19 Web Survey, which began fielding on March 13, 2020 (and last updated in our copy of the dataset on Feb 22, 2021) with daily random samples of the U.S. adults, aged 18 and older who are members of the Gallup Panel.

Results in the previous section showed that variables like the annual household income, gender, and political party play important roles in shaping individual-level acceptability of COVID-19 vaccination. Objective 2 aimed to determine the conditional effects between these variables on vaccine acceptance, and build a predictive model based on the conditional inference tree (CIT). The trained CIT model achieved high classification accuracy on the training set (75.5%), and is parsimonious, as shown in Fig 2. We also We also validated the model (since high accuracy on training set may just indicate model overfitting) by using randomly sampled 75% of the data points as training data, and using the remaining 25% as the test data. The accuracy of the model was found to be 73.7% when evaluated on this ‘withheld’ test set, illustrating robustness of the model to overfitting.

As illustrated in Fig 2, trust in the Trump administration, age, and ethnicity are the most important predictors. Only 34.9% participants had confidence that the Trump administration could successfully handle the pandemic. Again, consistent with OR shown in Table 2, those that trust in the administration are less likely to be vaccinated, and the younger female supporters of President Trump are the least likely to be vaccinated. However, older supporters with higher education or income responded more positively to the vaccine question.

For participants who did not trust in the administration, ethnicity is the strongest predictor. Black individuals were less likely to respond positively compared to White, Hispanic, Asian, and other ethnic individuals. Within the Black community, there are gender-specific differences. Men are predicted to be more accepting of the vaccine than women. A similar conclusion is also obtained for other ethnicities, but party, rather than gender, tends to be a stronger predictor. Democratic participants were significantly different from others, since more than 85% stated that they would agree to be vaccinated. Independents and Republicans were less positive, and again, compared to the older age-group, younger Independents and Republicans were less likely to say ‘yes’ to the vaccine question. We also experimented with some subjective well-being variables in the CIT model, but they were not found to play significant roles in predicting vaccine acceptance.

### Descriptive socio-demographic profiles of vaccine-hesitant U.S. adults

The *sum of odds ratios* gives us a descriptive score for each respondent’s vaccination willingness. The profile of those who have the highest sum of odds ratios turns out to be a male Democrat, more than 65 years old, who has a postgraduate degree, holds a full-time job or is not in the labor force (e.g., retired), has over $240,000 annual household income, and did not trust the Trump administration. The top 3 profiles correspond to such an individual who is of Asian, White and Hispanic ethnicity, respectively (in descending order). We found 14 respondents in our survey who fit one of these top 3 profiles, and we verified from their actual responses to the vaccine question that all of them were indeed willing to be vaccinated.

We also find the top three profiles who are the least likely to be vaccinated. The profile with the least sum-of-odds-ratios is a Black female Republican in the 41–54 age group, who has a part-time job with gross household income less than $60,000, and with less than a high school education. The next profile is the same as above, but the individual is White instead of Black. The third profile is the same as the first profile, except that the individual has a full-time job and is engaged in a technical, trade, vocational or business school or program after high school. Only three people were found to fit these (most vaccine hesitant) profiles, and none of them was willing to be vaccinated based on their actual response to the vaccine question.

These profiles are consistent with the core findings of the conditional inference tree, suggesting that the model is robust. Therefore, the odds-ratios score, in conjunction with the CIT, might be a useful tool for predicting how likely a *prototypical respondent* is to be vaccinated. For instance, if the demographic makeup of a local neighborhood is known (such as through census data), then the subgroups with high sum-of-odds-ratios (further validated by the CIT) may be vaccine hesitant and contributing to potential surges as new COVID-19 variants arise. As the predictive models and techniques improve further, public health officials may be able to use the predictions as a proxy for predicting COVID-19 hospitalizations and community surges. Such techniques may also help local, state and federal governments devise more effective communication and incentive strategies for meeting federal vaccination goals, which are currently falling short. We discuss this point further in the next section.

## Discussion

Despite major advances in vaccination, uptake of many vaccines continues to be suboptimal [38], including for COVID-19. The resurgence of vaccine-preventable illnesses has led the World Health Organization (WHO) to name vaccine hesitancy as one of the top ten threats to global health in 2019 [39]. To better understand the multidimensional nature of vaccine hesitancy, the WHO Strategic Advisory Group of Experts (SAGE) Working Group on Vaccine Hesitancy recommends a “3Cs” model, which highlights three intersecting categories of determinants: Confidence, Complacency, and Convenience [40]. However, many researchers argue that vaccine hesitancy could have more complex dimensions. For example, the specific social and political context can have significant effects on vaccine hesitancy [41, 42], and significant disparities in vaccine behaviors have also been observed across different socio-demographic groups [3, 5, 8].

This study adopted two different techniques to analyze the socio-demographic and partisan contexts of COVID-19 vaccine hesitancy. Similar to other work [25, 43], a univariate regression model was first applied to establish points of consistency with the findings of the global survey conducted by Lazarus et al. [5], while characterizing US-specific differences. The second method used was the conditional inference tree, which is a novel methodology for better understanding and interpreting the interaction effects of socio-demographic variables on the outcome (COVID-19 vaccine hesitancy).

The univariate regressions that were conducted suggest significantly different relative influence of different socio-demographic categories on vaccine acceptance in the U.S., compared to a previous global survey [5]. Concerning ethnicity, except for Asians, minorities are less likely to accept vaccination compared with White individuals. It is similar to Asian nations’ high vaccination acceptance shown in other work [5]. Additionally, the finding is consistent with other results shown in previous work: vaccine hesitancy was highest among Black individuals, in the UK, U.S., as well as in other nations [6, 7, 11, 13, 16, 20].

In considering the generalizability of our findings in an international context, we note that, although our objectives are aligned with some previous work [22–26, 42–44], the survey data used in our experiments spans a representative sample of adults across the entire United States, instead of focusing primarily on young adults [24, 43], or on a different country, such as UK [6, 7], Italy [23, 43] and Brazil [44]. Our core findings (associations between common socio-demographic variables, such as age and income, and vaccine hesitancy), including some of the findings implied by the conditional inference tree model, are consistent with these studies. A promising future area of research would be to apply the conditional inference tree model to data from these other countries to better understand its generalizability (and limits thereof).

People aged 41–54 are far less likely to accept the vaccine than those aged 18–24. This is also consistent with global survey results, but with one significant difference: people in the 25–64 age group are observed to be more vaccine accepting (compared to those aged 18–24) [5]. The vaccine acceptance of those aged 25–64 in our sample is smaller compared to younger people, especially for those aged 41–54. In the U.S., men are more vaccine-accepting than women, which is consistent with the findings in high-income countries or regions but different from the global survey results [5, 6, 15].

People at higher household income levels, as well as higher levels of education, are also more likely to be vaccine accepting, consistent with another U.S. vaccine hesitancy paper [16]. However, the consistency may only apply if we consider the national context: in other research looking at nations as a whole, evidence suggests that the vaccine acceptance rate in low- and middle-income countries was actually higher than in a high-income country, such as the U.S. [14].

We also found that people with a part-time job, or with an involuntary unemployed status, are less likely to positively respond to vaccination. Democrats are most inclined to vaccinate, while Republicans, Independents and also those from a third party are more likely to negatively respond to the vaccine question. Those who believed that the Donald Trump government could successfully manage emerging health challenges were less willing to be vaccinated. Finally, based on the conditional inference tree model, we find that among these socio-demographic correlates, trust in the administration, age, and ethnicity are the key predictors of vaccine hesitancy in the U.S. Trust in the Trump administration is the most important predictor. If people did trust the administration, then age is the second important to predict the vaccine acceptance of a U.S. adult. If a participant did not trust in the administration, the ethnicity then becomes the strongest predictor.

As suggested earlier, these responses could be used to devise effective communication strategies for targeting some identified vaccine-hesitant groups in local communities to get the vaccine. A recent article, for example, discusses how veterinarians and farmers (and through extension, people who tend to live in rural areas) share high levels of trust [45], and how this can be leveraged to reach people who do not trust ordinary sources of news. Similarly, others have pointed out the role that access and availability plays for people in low-income households to get the vaccine [46]. For the latter, vaccine acceptance could be improved simply by improving access, and through financial incentives to take the day off and get vaccinated, not dissimilar to other proposals for taking the day off to vote [47]. Among minority religious groups, church leaders can play an important role in encouraging people to get vaccinated, as an article last year illustrated [48]. Before implementing such measures, it is important to identify the local subgroups and communities that have high vaccine hesitancy, and to understand root causes at the local level. Our predictive models assist in the former task without requiring expensive individual polling in each community.

Among younger demographic groups in particular, social media has clearly emerged as a primary source of information, including for obtaining health information [49]. While still an evolving field, research into evidence-based social media interventions show that it could prove to be a promising way to counter misconceptions about vaccinations on the Internet [50–52], and promote greater uptake of vaccines [51, 53]. Such misinformation is rife on social media platforms, as several researchers have now shown. For example, according to Broniatowski et al. [50], content-polluting bots on social media were most likely to amplify anti-vaccination content whereas troll accounts tended to misrepresent their identities and purposefully instigate conflict between pro- and anti-vaccine users. Worryingly, anti-vaccine content frequently generated greater user engagement than its pro-vaccine counterparts, and widely shared across social media [52]. Unsurprisingly, exposure to such content has been found to directly influence vaccination opinions and cement vaccine hesitancy [54, 55]. In one study, Ahmed et al. [56] demonstrated that the use of social media, such as Twitter and Facebook, as sources of health information and knowledge, has a significant inverse association with vaccine uptake.

To counteract such messages and establish greater trust, some research [54, 57] suggests that healthcare providers should become more comfortable with social media platforms to better communicate with their patients. Similarly, health agencies and government websites should also aim to improve their overall social media presence, and try to foster partnerships with social media platforms [54, 58]. We note that there has been a general push among social media platforms [59, 60] to counteract health misinformation. For example, Pinterest [60] has redirected vaccine-related searches to a small set of carefully reviewed results from public health organizations, including the WHO and CDC. The platform has further disabled advertisements on these topics to prevent vaccine misinformation and the influence of external, nonscientific entities on matters of public health.

While this is a good first step, our results do suggest that different socio-demographic communities and users are vaccine-hesitant to different degrees. For example, a study showed that certain users are more susceptible emotional anti-vaccine posts, including those with cognitive impairment, older age, lower literacy, and less digital literacy [38]. For such groups, a more targeted and nuanced approach may be more productive, especially if the communication comes from a member of that community. Our conditional inference results may help to better identify and communicate with such communities.

### Limitations and future studies

There are some obvious limitations to the study that we hope to address in future work. First, because of the lack of relative data, we could not consider variables such as the job title/sector or the effects of the mass media in the regression and classification model. These are variables that likely also have strong impact on vaccine acceptance. Also, we did not study the changes in, or trends of, COVID-19 vaccine acceptance over time. This is an important agenda for future work, especially if it can be done in conjunction with limited causal analysis (such as changepoint detection techniques) to determine if there are variables that led to a rise or fall in vaccine hesitancy. Finally, as suggested in an earlier section, there is some evidence that spatial variables may have an influence on vaccine acceptance, although the political affiliation would also need to be controlled for in any such study. This is an interesting extension of the present study that we hope to pursue in future research.

## Conclusion

In this article, we assumed two objectives: first, to quantify the associations between common socio-demographic variables and vaccine acceptance in the U.S; second, to quantify the interaction and conditional effects of relevant socio-demographic variables, known to be important correlates of vaccine acceptance in the U.S., on vaccine acceptance.

In support of the first objective, univariate regressions allowed us to analyze in detail the relative effects of different categories of demographic variables on vaccine hesitancy. In support of the second objective, the conditional inference trees not only informed us about the importance of the variables but also reveals the conditional effects between these variables. Based on our results, we found that, while there is considerable response diversity in vaccine acceptance among different socio-demographic groups in the U.S., there were also clear patterns, some (but not all) of which were consistent with previous studies. Specifically, we found that trust in the Trump administration, age, and ethnicity are the most important factors to predict vaccine acceptance of American adults. In future work, we hope to further replicate our methods on survey data from other countries, and address the limitations and proposed extensions in the previous section.

## Supporting information

S1 Table Survey questions asked by Gallup to participants to obtain data for socio-demographic variables. (PDF)

S2 Table Description of participants and breakdown of the COVID-19 vaccine acceptance questions. (PDF)

S3 Table The numeric values supporting the data in Fig 1. (PDF)

S4 Table STROBE checklist. (PDF)

S1 Text Sampling methodology used by Gallup (relevant extract quoted verbatim). (PDF)
