# Supplementary material for: Using conditional inference to quantify interaction effects of socio-demographic covariates of US COVID-19 vaccine hesitancy
Source: PLOS Glob Public Health. 2023 May 12;3(5):e0001151. doi: 10.1371/journal.pgph.0001151 (PMC10180637; doi:10.1371/journal.pgph.0001151)
Supplement: S1 Table — (PDF) [file pgph.0001151.s001.pdf]

S1 Table: Survey questions asked by Gallup to participants to obtain data for socio-demographic variables

Annual household income	What is your total annual household income before taxes?
	1 Less than $12,000
	2 $12,000 to $23,999
	3 $24,000 to $35,999
	4 $36,000 to $47,999
	5 $48,000 to $59,999
	6 $60,000 to $89,999
	7 $90,000 to $119,999
	8 $120,000 to $179,999
	9 $180,000 to $239,999
	10 $240,000 and over
Employed	Which of the following best describes your situation?
	1 Employed full-time
	2 Employed part-time
	3 Retired
	4 A homemaker
	5 A full-time student
	6 Unemployed but looking for work
	7 Unemployed and not looking for work
party	In politics, as of today, with which political party do you most closely affiliate?
	1 Democrat
	2 Republican
	3 Independent
	4 Other party
trust in Trump administration	Please think about the recent impact of the coronavirus (COVID-19) on your life when responding to the following and indicate your level of agreement or disagreement.
	I have confidence in the leadership of President Donald Trump to successfully manage emerging health challenges.
	5 Strongly agree
	4
	3
	2
	1 Strongly disagree
vaccine acceptance	If an FDA-approved vaccine to prevent coronavirus/COVID-19 was available right now at no cost, would you agree to be vaccinated?

	1. Yes
	2. No

Note that since gender, age, race, Hispanic ethnicity, education, and region are required information for participants, Gallup directly asks participants to select the option that best describes themselves.
