# Supplementary material for: Using conditional inference to quantify interaction effects of socio-demographic covariates of US COVID-19 vaccine hesitancy
Source: PLOS Glob Public Health. 2023 May 12;3(5):e0001151. doi: 10.1371/journal.pgph.0001151 (PMC10180637; doi:10.1371/journal.pgph.0001151)
Supplement: S2 Table — (PDF) [file pgph.0001151.s002.pdf]

S2 Table: Description of participants and breakdown of the COVID-19 vaccine acceptance questions

	overall
n	16,322
Gender (%)	
Female	7,429 (45.52)
Male	8,893 (54.48)
Household income level (%)	
[0, 36,000)	2,065 (12.65)
[36,000, 60,000)	2,711 (16.61)
[60,000, 90,000)	3,279 (20.09)
[90,000, 120,000)	3,021 (18.51)
[120,000, 180,000)	2,847 (17.44)
[180,000, 240,000)	1,143 (7.00)
[240,000,∞)	1,256 (7.70)
Education level (%)	
Less than high school	59 (0.36)
High school	1,313 (8.04)
Bachelor’s degree	3,885 (23.80)
Postgraduate degree	4,129 (25.30)
Other	6,936 (42.49)
Age group in years (%)	
18-24	82 (0.50)
25-40	2,273 (13.93)
41-54	3,412 (20.90)
55-64	3,844 (23.55)
65+	6,711 (41.12)
Party (%)	
Democrat	6,822 (41.80)
Republican	4,105 (25.15)
Independent	5,002 (30.65)
Other	393 (2.41)
Ethnicity (%)	
White	14,318 (87.72)
Asian	270 (1.65)
Black	717 (4.39)
Hispanic	836 (5.12)

Other	181 (1.11)
Employment status (%)	
Full-time	6,932 (42.47)
Part-time	1,393 (8.53)
Involuntary-unemployed	606 (3.71)
Not-in-labor-force	7,391 (45.28)
Trust in Trump administration (%)	
Yes	5,703 (34.94)
No	10,619 (65.06)
Accept COVID-19 vaccine if generally available (%)	
Yes	11,616 (71.17)
No	4,706 (28.83)
