# Supplementary material for: Using conditional inference to quantify interaction effects of socio-demographic covariates of US COVID-19 vaccine hesitancy
Source: PLOS Glob Public Health. 2023 May 12;3(5):e0001151. doi: 10.1371/journal.pgph.0001151 (PMC10180637; doi:10.1371/journal.pgph.0001151)
Supplement: S3 Table — (PDF) [file pgph.0001151.s003.pdf]

S3 Table: The numeric values supporting the data in Figure 1

state (compared to CA)	Odds Ratio	p-value	[95% Conf. Interval]	
AK	1.065799	0.829	0.5987276	1.897237
AL	0.6572922	0.008	0.4812886	0.8976589
AR	0.5635371	0.003	0.3850351	0.8247926
AZ	0.7683359	0.024	0.6112512	0.9657895
CO	0.9268537	0.541	0.7264987	1.182463
CT	0.8408332	0.278	0.6148142	1.149942
DC	3.688724	0.002	1.584593	8.586864
DE	1.560149	0.21	0.7779672	3.128749
FL	0.8067365	0.018	0.6749284	0.9642856
GA	0.7065545	0.002	0.5662803	0.8815762
HI	2.757472	0.034	1.079148	7.045975
IA	0.954803	0.778	0.691893	1.317616
ID	0.607338	0.01	0.4154843	0.8877818
IL	1.031318	0.779	0.8316641	1.278903
IN	0.8857205	0.346	0.687995	1.140271
KS	0.9622757	0.832	0.6750072	1.371799
KY	0.7502489	0.086	0.5406653	1.041076
LA	0.5200496	0	0.3680989	0.7347253
MA	1.661059	0.001	1.243422	2.218972
MD	0.8773775	0.296	0.6866337	1.121109
ME	0.9938258	0.98	0.6097009	1.619958
MI	0.8633653	0.2	0.689524	1.081035
MN	1.344588	0.026	1.035719	1.745567
MO	0.7090072	0.008	0.5505249	0.9131126
MS	0.4768561	0.001	0.3035535	0.7490993
MT	0.5555762	0.012	0.3516562	0.8777463
NC	0.843112	0.136	0.6737898	1.054985
ND	1.209418	0.62	0.5701086	2.565636
NE	1.030754	0.869	0.7199677	1.475695
NH	1.088476	0.773	0.6123711	1.934741
NJ	0.9317353	0.549	0.7394498	1.174022

NM	0.9252045	0.688	0.6326902	1.352958
NV	0.8843866	0.57	0.5785952	1.351791
NY	0.9924817	0.936	0.8247963	1.194258
OH	0.7974182	0.029	0.6505478	0.9774467
OK	0.6802974	0.019	0.4937175	0.9373873
OR	0.9456616	0.673	0.7296118	1.225687
PA	0.8965816	0.251	0.7441797	1.080194
RI	1.917791	0.117	0.8493555	4.33025
SC	0.8981413	0.504	0.655539	1.230526
SD	0.8372891	0.597	0.4331895	1.618352
TN	0.7040966	0.009	0.5421026	0.9144985
TX	0.7969573	0.007	0.6764338	0.9389549
UT	1.040099	0.805	0.761648	1.420349
VA	1.13546	0.252	0.9137424	1.410978
VT	0.9675341	0.914	0.5297317	1.767163
WA	0.9594172	0.706	0.7737477	1.18964
WI	0.8375499	0.164	0.6527002	1.074751
WV	0.9357073	0.809	0.5454297	1.605245
WY	0.4201135	0.006	0.2254306	0.7829253
