# Supplementary material for: Using conditional inference to quantify interaction effects of socio-demographic covariates of US COVID-19 vaccine hesitancy
Source: PLOS Glob Public Health. 2023 May 12;3(5):e0001151. doi: 10.1371/journal.pgph.0001151 (PMC10180637; doi:10.1371/journal.pgph.0001151)
Supplement: S4 Table — (PDF) [file pgph.0001151.s004.pdf]

S4 Table: STROBE checklist

STROBE Statement—Checklist of items that should be included in reports of ***cross-sectional studies***

	Item No	Recommendation
**Title and abstract**	1	☑ ~~(*a*) Indicate the study's design with a commonly used term in the title or the abstract~~
		☑ ~~(*b*) Provide in the abstract an informative and balanced summary of what was done and what was found~~
**Introduction**		
Background/rationale	2	☑ ~~Explain the scientific background and rationale for the investigation being reported~~
Objectives	3	☑ ~~State specific objectives, including any prespecified hypotheses~~
**Methods**		
Study design	4	☑ ~~Present key elements of study design early in the paper~~
Setting	5	☑ ~~Describe the setting, locations, and relevant dates, including periods of recruitment, exposure, follow-up, and data collection~~
Participants	6	☑ ~~(*a*) Give the eligibility criteria, and the sources and methods of selection of participants~~
Variables	7	☑ ~~Clearly define all outcomes, exposures, predictors, potential confounders, and effect modifiers. Give diagnostic criteria, if applicable~~

Data sources/ measurement	8*	☑ ~~For each variable of interest, give sources of data and details of methods of assessment (measurement). Describe comparability of assessment methods if there is more than one group~~
Bias	9	☑ ~~Describe any efforts to address potential sources of bias~~
Study size	10	☑ ~~Explain how the study size was arrived at~~
Quantitative variables	11	☑ ~~Explain how quantitative variables were handled in the analyses. If applicable, describe which groupings were chosen and why~~
Statistical methods	12	☑ ~~(*a*) Describe all statistical methods, including those used to control for confounding~~
		☑ ~~(*b*) Describe any methods used to examine subgroups and interactions~~
		☑ ~~(*c*) Explain how missing data were addressed~~
		☑ ~~(*d*) If applicable, describe analytical methods taking account of sampling strategy~~
		☑ ~~(*e*) Describe any sensitivity analyses~~
**Results**		
Participants	13*	☑ ~~(a) Report numbers of individuals at each stage of study—eg numbers potentially eligible, examined for eligibility, confirmed eligible, included in the study, completing follow-up, and analysed~~
		☑ ~~(b) Give reasons for non-participation at each stage~~
		☐ (c) Consider use of a flow diagram

Descriptive data	14*	☑ ~~(*a*) Give characteristics of study participants (eg demographic, clinical, social) and information on exposures and potential confounders~~
		☐ (b) Indicate number of participants with missing data for each variable of interest
Outcome data	15*	☑ ~~Report numbers of outcome events or summary measures~~
Main results	16	☑ ~~(*a*) Give unadjusted estimates and, if applicable, confounder-adjusted estimates and their precision (eg, 95% confidence interval). Make clear which confounders were adjusted for and why they were included~~
		☑ ~~(*b*) Report category boundaries when continuous variables were categorized~~
		☑ ~~(*c*) If relevant, consider translating estimates of relative risk into absolute risk for a meaningful time period~~
Other analyses	17	☑ ~~Report other analyses done—eg analyses of subgroups and interactions, and sensitivity analyses~~
**Discussion**		
Key results	18	☑ ~~Summarise key results with reference to study objectives~~
Limitations	19	☑ ~~Discuss limitations of the study, taking into account sources of potential bias or imprecision. Discuss both direction and magnitude of any potential bias~~
Interpretation	20	☑ ~~Give a cautious overall interpretation of results considering objectives, limitations, multiplicity of analyses, results from similar studies, and other relevant evidence~~

Generalisability	21	☑ ~~Discuss the generalisability (external validity) of the study results~~
**Other information**		
Funding	22	☑ ~~Give the source of funding and the role of the funders for the present study and, if applicable, for the original study on which the present article is based~~

*Give information separately for exposed and unexposed groups.
