# Supplementary material for: Using conditional inference to quantify interaction effects of socio-demographic covariates of US COVID-19 vaccine hesitancy
Source: PLOS Glob Public Health. 2023 May 12;3(5):e0001151. doi: 10.1371/journal.pgph.0001151 (PMC10180637; doi:10.1371/journal.pgph.0001151)
Supplement: S1 Text — (PDF) [file pgph.0001151.s005.pdf]

S5: Sampling methodology used by Gallup (relevant extract quoted verbatim)

“The COVID-19 web survey began fielding on March 13, 2020 with daily random samples of U.S. adults, aged 18 and older who are members of the Gallup Panel. Approximately 1,200 daily completes were collected from March 13 through April 26, 2020. From April 27 to August 16, 2020 approximately 500 daily completes are being collected. Starting August 17, 2020, the survey moved from daily surveying to a survey conducted one time per month over a two week field period (typically the last two weeks of the month).

The Gallup Panel is a probability-based, nationally representative panel of U.S. adults. Members are randomly selected using random-digit-dial phone interviews that cover landline and cellphones and address-based sampling methods. The Gallup Panel is not an opt-in panel.

The data are weighted to minimize bias in survey-based estimates. The sample for this study, as described in the previous sections, utilizes the Gallup panel. The Gallup panel maintains weights for all members that are based on their selection probabilities. These weights were used as base weights for weighting the final dataset of completes.

The weighting starts with the panel base weight as the initial weight. Base weights take into account the probability of selection into the panel for all stages of selection. Next, post-stratification weights are created to adjust for non-response bias. Targets for post-stratification weighting are generated from the 2017 Current Population Survey (CPS). Nonresponse adjustments are made by adjusting the sample to mat
